# Compensation of Physicians Especially in the Western States by Dr. Drake

**Published:** 1838

**Authors:** 


### Art. V.—Compensation of Physicians.

*Observations on the compensation of Physicians especially in the Western States.* By one of the Editors.

Our efforts to obtain a list of the fees of our medical brethren, from such a number of points in the Valley of the Mississippi as would afford a fair exhibit of their rates of charging, have not been so succesful, as to enable us, in this number, to present a tabular view. We respectfully solicit further contributions, especially from the states south of the Ohio river; and propose, mean while, to devote a few pages to the discussion of some general principles involved in the compensation of medical practitioners.

The people, at large, rather than those who practise medicine, are interested in the learning, skill and devotedness of the profession; for experience has shown, that in the absence of the whole, the confidence of the community can be acquired, even to a more profitable degree, than by their aid. If a colony of people were about to cross the gulph of Mexico, would it not be absurd for them to say, let the pilots, engineers and sailing masters look to their own qualifications and dignity, we have no concern in those matters? But suppose, still further, that those who guided and impelled the ships, were protected by supernatural or any other means, from the dangers of the sea, while the passengers were not, would it not be ridiculously absurd, for the latter to say, we shall not trouble ourselves about the scientific and nautical skill of those who are to take charge of us, that is their own affair? All the world would answer this question in the affirmative; and yet, this same world, with a few exceptions, perpetrates an absurdity quite as great as this, in reference to the medical profession. It is passing strange, that those who are to suffer under the mistakes of ignorance, heedlessness, and profligacy, should be the last to scan the character of the men whom they employ.

Dim, indeed, is the eye of common sense, in any people, when it cannot perceive, that the physician should be familiarly acquainted with several sciences, all of which, except anatomy and chemistry, are imperfect and speculative; and that, in his practical duties, he is to apply the half formed rules deduced from these sciences, to the abatement of disease, and the restoration of health—duties which require patient observation, sound judgment and deep penetration. For these to be properly developed, a long and laborious course of study is indispensable, and the taste and temperament of the individual must be moulded in a specific manner. His vocation is therefore incompatible with any other pursuit. Still further, the physician should be, at *all times*, intellectually and physically prepared, to execute whatever service may be suddenly thrown upon him. How then can he, in justice, to the community, abstract his thoughts from his professional duties, and direct them on any other business? As easily might he serve both God and mammon—as soon direct the movements of the ship in battle, and at the same time amputate the limbs of the wounded.

The genius of the medical profession, excludes then, all other callings, as *means of support;* it claims the practitioner, both soul and body, as its own; commands him not to wander into the paths of commerce, the mechanic arts or pecuniary speculation; and tells his own conscience and the community, if he should, that he lives in the violation of duty. At the same time, no other profession involves its members in such extensive and varied social relations. In every part of the United States, the physician is, or ought tobe, a person of high relative rank—he should be a man of letters and science—a gentleman in habits and manners. He cannot, therefore, live in retirement and poverty. Like an actor while the play is advancing, he must be ready at any moment to enter upon the stage, and is obliged to appear in the costume appropriate to his character.

From these premises it results, that the compensation of the physician should be liberal. It ought to bring back with interest, what had been expended in the acquisition of elementary knowlege, and afford compensation for the tedious years of probation, to which he was subjected; it should enable him to procure all the necessary books, apparatus and instruments; provide adequately for the support and education of his family ; and secure him against want in his old age.

If medical practitioners are not rewarded to this extent, their fees are too low. We know of no other standard. This is their just demand. The claim of the profession upon society. The only condition on which the former can be kept prepared to administer, successfully, to the wants of the latter. If society would, then, provide itself with the best medical aid which is practicable, it must pay the price which such aid *necessarily* costs. It must class medical advice and medicines among its natural wants, and draw for their relief, out of this same treasury whence it pays for bread and meat. These ideas, however, are foreign to the minds of the majority. They call sickness misfortune, and regard the expenditure of money, for its relief, as augmenting their calamities. Hence they are forever at war, with those whom they invoke in the hour of bodily distress. Many have nothing to pay, and still claim the services of the profession, with an impudence which is but the exponent of the views of society at large.—Others, and the number is not a few, consider it in no degree dishonorable, to neglect payment for an indefinite time, and finally escape from it by fraud. Others assume the privilege of postponing the claims of their physicians to those of all other creditors. Finally, all classes, from the pauper to the nabob, combine in denunciations at the rate of charging, *whatever it may be*, which prevails among them.

All this reacts powerfully on the happiness of society, by its pernicious influence on a profession, which has much more to do with that happiness, than most other callings can have.

The want of a more liberal and willing compensation, compells a great number of our physicians, to add some other *business* to the practice of the profession; while many are tempted to abandon it altogether, for more lucrative employments. Of those who are not driven into either of these measures, not a few become disgusted with a calling, which exacts from them sleepless application, and fails to make any opulent return—a state of feeling little favorable to mental improvement. Lastly, when the natural disposition of the practitioner is in any degree avaricious, his slender compensation is apt to generate oppression of the poor. Such are the deteriorating effects of inadequate compensation.

The honest earnings of medical men exceed, to an incredible degree, their actual receipts; because they are never without patients, of whom they cannot demand any thing. But whence arises the claim of pauperism on the medical profession?—We can perceive no obligation resting on the physician to give his services, gratuitously, to a poor family, that is not binding on all who surround such a family, to supply it with the necessaries and comforts of life. The judgment of society on this point is arbitrary and selfish. An upright decision would be, that in proportion as the time and energies of a physician are expended upon the poor, his rate of charging the rich, should be increased. Such, however, is not the opinion of the latter, because their interest controls their judgment and conscience. But of those against whom the physician does make charges, a great proportion fail to pay him. Some become insolvent, others emigrate and are lost sight of, others dispute his prices and the aggregate amount, compelling him to abandon his claims, or to prosecute their collection in the courts of justice; finally, public sentiment prohibits a resort to the law, under a variety of circumstances, in which it would be tolerated in other creditors. For example, the physician who should sue his patient when he had just recovered from an illness, would bring censure on himself and the profession; but the merchant or grocer, who might do the same thing, for necessaries in that sickness, would pass unreproved. We again ask, where is the justice of this distinction? If the rich sympathize with the poor when they see payment exacted, by the medical men who have alleviated their sufferings and saved their lives, then, we say let the rich step forward and equallize the burden, by distributing it among themselves, until each of them has the proportion which is left to the physician. This would be far more honorable, than to pour out sneers and obloquy. They should "come into court with clean hands"—their own charities should be made liberal, before they have a right to pass sentence upon those of their neighbors.

The practice of attending the poor gratuitously, and those in moderate circumstances for a slender and uncertain, occasional, compensation, is, however, universal, and not likely to cease.—Such being the case, it follows, that the support of a physician must be derived from those who are in comfortable or opulent circumstances; and having made the amount of property an element in the system of charging we should carry it out. If, according to public opinion, the patient who has not the means of paying *much*, should be charged but little, it follows, that the patient who is wealthy should be charged a great deal. The rule should work both ways. It does not, however, work upward without resistance. The rich man says—you should attend the poor gratuitously, but you must charge me no more than the mechanic who administers to my artificial wants! Admirable consistency!—The impudent offspring of a cold-hearted selfishness. There are three strata in society, the rich, the poor and the intermediate, which graduate each other. The charges of a physician should be framed with a reference to the last. As he descends among the poor, they must decrease till they vanish; as he ascends to the rich, they should be increased, in proportion to their reduction in the other direction. This is JUST, *because* it is but carrying out the conventional idea, that poverty ought to modify our charges. It assumes that if poverty should reduce—riches should augment them—an assumption which cannot be overthrown. We ought to look to our rights in this matter. In every town it should be the practice of the physicians, to charge higher as their patients are wealthier; and they should sustain each other in the collection of their claims, on this principle.

Our charges ought to be modified by another circumstance.—In chronic cases, obstinate but not violent, requiring occasional visits for a great length of time, the price charged for each, should be greater, than in cases which require one or two visits, *per diem.* A foundation for this rule exists in the extent of the inquiries and renewed examinations, which are necessary, to reproduce in the mind of physician, the exact condition of his patient a week before, and thus enable him to compare the present with the past.

Again. The nature of the disease and the temper of the patient should modify the rate of charging. When detained an unusual legth of time, or required, habitually, to make his visit at a specific hour, to the neglect of other business, a greater sum ought to be exacted.

Again. When a disease is protracted far beyond its ordinary duration, or one that is generally cured, proves fatal, our bills should, in most instances, be rendered with some abatement; while on the other hand, when that, which is usually protracted, is cut short, or that which commonly proves fatal, is cured, the reward ought to be greater, than should be claimed, for the same amount of personal attendance, in ordinary cases. This principle does, indeed, obtain extensively in our surgical practice and why should it not in our medical? The reward for operations, is in proportion to the results obtained, not the extent of cutting; in the practice of physic it *should* be the same, but is not. Very often, by ten visits the life of an individual is saved, and still, only ten, more likely but five, dollars are charged; while, for an operation that should preserve life, a hundred would be regarded as below the mark. Such a distinction between two branches of the same profession, is unwarrantable and absurd.

Finally. Experienced and aged physicians, should charge higher than the younger. The latter, as a general remark, are less efficient, and their patients commonly poorer. When members of the Bar, of different ages and grades of talent and experience, are associated in the same cause, by the common sense of all concerned, the young are paid less than the older and abler. Why should not the same rule apply to physicians? As they advance in age and character, they should place a higher value on their services, and sustain each other in collecting on this principle. The younger members of the profession are much interested in this matter, for as long as the community can obtain the suggestions of ripe experience, as cheap as the theoretical prescriptions of youth, they are likely to prefer the former. The physician of 40, who is afraid to advance his charges, lest he should lose his business, discloses that he is conscious of having grown in years faster than in knowledge. Such an one has found his way into the ranks of the profession by some mistake—his destiny has been violated.

Throughout the Valley of the Mississippi the practice of extended credits to our patients, is a great inconvenience and frequently a serious loss. Many who could pay, disappear before the time when, according to custom, their bills may be sent in; and, in accordance with the deepest principles of human nature, the disposition to discharge them decreases with the lapse of time from the illness. This disposition is the highest guaranty which the physician has, and fashion should not counteract its advantages to him. Still further. A vicious public opinion has decreed, that the patient, after his recovery, may answer to and reinstate himself with all the world, before he compensates the very man who has preserved him to that world. Common sense and common justice, could they be allowed to speak, would pronounce, that the man who achieves this, should be rewarded first. We need not say how seldom this is done.

It may be asked, whence all this grumbling? Is not the medical, a charitable profession? Does it not even boast of its benefactions? Where is its vaunted dignity, when it condescends to complain of its rewards? We may answer these questions, by asking others. What gave to the profession this high character, but the charities it always has and will administer, beyond every other? But are physicians the agents of society in these administrations? We say no: Only a part of them are even known to the public. Is the physician a stipendiary of the state? Does he enjoy any specific immunities? The answer is still in the negative. In Ohio and some other states, his profession is even taxed. Must he not pursue it, then, as a business—a source of support for himself and his family? And in doing this, must not the rules of business govern both him and those who seek his services? The great amount of his private charities, renders it the more necessary for him to be adequately and promptly compensated by those who are able to pay him. No considerations of delicacy should deter him from seeking this compensation—no sophistry mistify his mind on this point. He is not less worthy of his hire than those who wield the pen or axe, nor more capable of living and laboring without reward, than they. Thus while he bestows liberal charities on the indigent, his relations with those who have property, must be governed by the rules which regulate the business of the country.

If the profession in the West, were paid more willingly and adequately, it would speedily rise to higher usefulness. Boys of greater promise and ampler preparation would be put to the study. The stimulus of prospective wealth would be added to that of promised fame; and the number of aspirants for the latter would be increased, by the influence of the former. The governing principle of the age is the love of money; but this principle *may* be united with those of a milder and holier temperament. Thus softened it does no harm but much good, both to the individual and the community. If the rewards of the profession in the Valley of the Mississippi were doubled, its scientific respectability would rise in the same proportion, and its charities suffer no abatement.

But without the co-operation of society, this means of raising it into higher skill and usefulness, cannot be made available. It depends, then, on the people themselves, whether they will confide the preservation of their limbs and lives to the illiterate and superficial, or to men deeply read in the philosophy of the human body, and accurately acquainted with the means of removing its disorders—the former are a curse, the latter a blessing to the world.

